# Hospital Clothing and Bedding

**Published:** 1888-09-15

**Authors:** G. Jaeger


### HOSPITAL CLOTHING AND BEDDING.

THE point of most consequence in hospital clothing is not the clothing of the nurses, but the clothing and bedding of the patients, and the nature of the furniture and walls of the room. If the former be all of pure wool, and the furniture be either of iron, or so painted or varnished as to be wholesome ; further, if the walls are treated in the manner which I will subsequently explain, two eminent advantages will be achieved :—

1. Patients will sooner rid themselves of the poison of their disease, and will therefore sooner be cured, for the same holds good of disease-poisons as of the attenuated self-poison of healthy people. The power of attraction which plain wood and dead plant-fibre possess for evil odours prevents the latter's escape according to the laws of diffusion, and defeats in great part, if not altogether, endeavours to ventilate.

2. These measures minimise the chief of the two conditions necessary to infection, namely, the tendency to disease, a consideration which is especially important, because the great facility with which disease-germs are conveyed renders the most careful rules insufficient to prevent their transport.

As regards the clothing of nurses, in order that it may not collect the human poisons which are generated in sick-rooms and that the nurses themselves may be proof against epidemics, they should be clothed in wool throughout. This clothing should be protected (but only when they are actually nursing, and the clothing is liable to be soiled by the excretions of the patients) by a cotton or linen cover, which should be washed daily.

Lastly, as to the walls of rooms. Many authorities on hygiene attach much value to the so-called porous ventilation, *i.e.*, to the fact that ordinary walls are pervious to the air, and thus ensure a certain amount of ventilation. Covering the walls of a room with oil-paint is therefore condemned as shutting off this means of ventilation. I formerly held the same view, and caused my walls to be distempered ; but since I have tried the other plan, I have altered my opinion. I first caused my staircase, in which there was always a smell from the water-closet, to be treated with oil paint, with the result of greatly improving the freshness of the air, and I have subsequently painted other portions of the interior of my house with a similar satisfactory effect. The explanation is very simple : the relations of plaster, lime, and stone to odours are similar to those of plain wood ; through their surface-attraction they hinder the egress of mal-odorous exhalations from living beings. When the walls are unpainted, and especially when they are papered, the air cannot be pure, and the ventilation which takes place through the by no means fragrant materials of the walls is nothing to be grateful for. Oil paint destroys the evil influence, and offensive odours pass away into the outer air much more freely than before, even without any special ventilating contrivance.

Let the experiment be tried, in some one room in a hospital, of coating walls, flooring, and all woodwork and wooden furniture thoroughly with oil paint, not merely the parts which are seen, but inside, outside, and all round ; let the bedding and clothing of the patients be wholly of pure wool, and then estimate the result by the evidence of the nose ! In such a "normal" hospital ward the atmosphere will, under equal conditions of ventilation and cleanliness, be distinctly better than that in ordinary wards. G. JAEGER, M.D.

